# Prophage Genomics and Ecology in the Family *Rhodobacteraceae*

**DOI:** 10.3390/microorganisms9061115

**Published:** 2021-05-21

**Authors:** Kathryn Forcone, Felipe H. Coutinho, Giselle S. Cavalcanti, Cynthia B. Silveira

**Affiliations:** 1 Department of Biology, University of Miami, 1301 Memorial Dr., Coral Gables, Miami, FL 33146, USA; kathryn.forcone@miami.edu (K.F.); gscanti@gmail.com (G.S.C.); 2 Evolutionary Genomics Group, Departamento de Producción Vegetal y Microbiología, Universidad Miguel Hernández de Elche, Aptdo. 18, Ctra. Alicante-Valencia, s/n, 03550 San Juan de Alicante, Spain; felipehcoutinho@gmail.com; 3 Department of Marine Biology and Ecology, Rosenstiel School of Marine and Atmospheric Sciences, University of Miami, 4600 Rickenbacker Causeway, Miami, FL 33149, USA

**Keywords:** roseophage, auxiliary metabolic genes, global distribution

## Abstract

Roseobacters are globally abundant bacteria with critical roles in carbon and sulfur biogeochemical cycling. Here, we identified 173 new putative prophages in 79 genomes of *Rhodobacteraceae*. These prophages represented 1.3 ± 0.15% of the bacterial genomes and had no to low homology with reference and metagenome-assembled viral genomes from aquatic and terrestrial ecosystems. Among the newly identified putative prophages, 35% encoded auxiliary metabolic genes (AMGs), mostly involved in secondary metabolism, amino acid metabolism, and cofactor and vitamin production. The analysis of integration sites and gene homology showed that 22 of the putative prophages were actually gene transfer agents (GTAs) similar to a GTA of *Rhodobacter capsulatus.* Twenty-three percent of the predicted prophages were observed in the TARA Oceans viromes generated from free viral particles, suggesting that they represent active prophages capable of induction. The distribution of these prophages was significantly associated with latitude and temperature. The prophages most abundant at high latitudes encoded *acpP*, an auxiliary metabolic gene involved in lipid synthesis and membrane fluidity at low temperatures. Our results show that prophages and gene transfer agents are significant sources of genomic diversity in roseobacter, with potential roles in the ecology of this globally distributed bacterial group.

## 1. Introduction

Prophages (phage genomes integrated into bacterial genomes) represent a major driving force in bacterial evolution due to genomic rearrangements and the diversification of genetic repertoires [1,2,3,4,5]. Half of the fully sequenced bacterial genomes contain at least one prophage and prophage-encoded genes can represent up to 35% of a bacterial species pangenome [2,6,7]. Over time, prophages can lose their ability to induce and become genomic islands domesticated by the bacterial hosts [1]. These defective prophages commonly encode genes that have been coopted by the bacterial hosts for other functions, such as cell communication and warfare [8]. The changes in bacterial metabolism and ecological interactions caused by active and defective prophages have the potential to impact the biogeochemical and ecological roles of globally abundant bacterial groups.

*Rhodobacteraceae* can comprise up to 36% of the bacterial community in marine habitats such as the upper mixed layer of the ocean [9,10,11]. The *Rhodobacteraceae* family includes members of the *Roseobacter* genus and is historically referred to as the roseobacter group [12]. Sulfur metabolism and aerobic anoxygenic photosynthesis catalyzed by roseobacters impact global carbon and sulfur biogeochemical cycles [13,14,15]. Roseobacters provide vitamin B_12_ to B_12_-auxotrophic eukaryotes including diatoms, dinoflagellates, and coccolithophores, impacting primary production at large scales [16,17,18]. Although most roseobacters are marine, many representatives inhabit soils, freshwater lakes, and hypersaline habitats [19]. Their abundance and ubiquity are largely attributed to their genome plasticity, characterized by streamlined genomes and multiple extrachromosomal replicons [9,20,21]. Genes encoded by extrachromosomal elements modulate traits such as the production of antibiotics, secretion systems, and the ability to switch between planktonic and biofilm growth forms [14,21,22]. The presence of ecologically relevant genes in extrachromosomal elements suggests that lateral gene transfer has a pivotal role in the ecology of roseobacters.

The viral infection of roseobacters has been hypothesized to regulate the biogeochemical roles of this group [23]. Viruses influence oceanic nutrient and biogeochemical cycles, population dynamics, and bacterial densities [24,25]. The effect that viruses have on their hosts’ ecological roles depends on their mode of infection. Lytic predator–prey interactions can control bacterial population densities and regulate the rate of biomass and organic matter transformations [26,27]. In temperate interactions such as lysogeny, the phage integrates into the host’s genome as a prophage or replicates as an extrachromosomal element [28,29]. Few genes are normally expressed during the lysogenic state, but these genes can modify bacterial phenotypes and the bacteria’s interactions with its environment and other community members [30]. Some examples are the protection against infection by other phages (superinfection exclusion), protection against phagocytosis by eukaryotes, and transfer of auxiliary metabolic genes (AMGs) [31,32,33,34,35,36,37].

The first marine phage to have its genome completely sequenced was a roseophage, SIO1 [38]. Since this first description, only 34 roseophages were isolated [23,39,40]. Sixty-eight percent of them belong to the family *Podoviridae* and 27% to *Siphoviridae* within the order *Caudovirales*, and 5% to *Microviridae* [23,40,41,42,43]. More recently, two roseophages were isolated from the North Sea and classified in the novel Cobavirus sub-group within the family *Podoviridae* [44]. Two cultivated phages (SIO1 and P12053L) and six metagenome-assembled phages form a genus-level phylogenetic clade with the Cobaviruses according to their amino acid sequence similarity [44]. N4-like phages are another recently described group of roseophages initially classified as *Podoviridae* and likely to be placed in their own family, *Schitoviridae* [45,46,47,48]. N4-like phage genomes contain a set of 14 core genes with functions in DNA replication and transcription, including RNA polymerases [46,47,49]. Members of the N4-like roseophages encode the auxiliary metabolic genes thioredoxin (*trx*) and ribonucleotide reductase (*rnr*), a gene involved in nucleotide metabolism that is widespread in marine phages [46,50,51].

The modification of roseobacter metabolism by phage infection has been demonstrated in three species [39,52]. The lytic phage infection of *Dinoroseobacter shibae* and *Roseobacter dentrificans* changes the global host gene expression pattern and proteome [39,52]. Some of the functions most notably affected are nucleotide metabolism, ion metabolism, and replication and repair [52]. Proteins that exhibited changes in expression in response to the infection of *Roseobacter dentrificans* include a transcription antitermination protein, a putative sugar transporter, and putative ribosomal proteins [39]. Another example is the lytic infection of *Sulfitobacter* sp., which alters carbon and nitrogen partition in the bacterial biomass and lysate due to the differential incorporation of these elements into phage particles [53]. These data obtained from isolated roseophages suggest that phage infections have the potential to significantly impact the global biogeochemical roles of roseobacter.

Of the 34 isolated roseophages, only 5 are identified as temperate. These phages were isolated by plaque assay or Mitomycin C induction from *Roseovarius nubinhibens*, *Thiobacimonas profunda*, *Pelagibacter abyssi*, and *Sulfitobacter* sp. Strain 2047 [40,44,54,55,56]. Among the isolated roseophages identified as lytic in their original description, 75% contain an integrase or lytic repressor gene that are characteristic of temperate phages, suggesting that they may have the ability to integrate, although this has not been observed in laboratory conditions [40]. Interactions between temperate phages have been reported to occur in roseobacters, with impact on host physiology [57]. Superinfection of the lysogenic *Sulfitobacter* sp. Strain 2047 by a genetically similar phage produces phages from both lytic infection and prophage induction, and modifies the host gene expression [57]. Such multi-species interactions may be pronounced during the large increases in roseobacter abundance, as indicated by a large change in roseobacter-encoded transposases during a phytoplankton bloom [58].

Here, we investigated the genomics and ecology of prophages integrated in the genomes of 79 bacterial species representative of the roseobacter group [59]. Our data show that roseobacter prophages are host-specific, with the exception of one *Microviridade* prophage that was found in three roseobacter species. Sequences with high similarity to the newly identified prophages were present in metagenomes from the free viral fraction of seawater samples, indicating that they represent active prophages capable of induction. Our study shows that globally abundant prophages encode auxiliary metabolic genes that likely impact their host metabolism and ecology.

## 2. Methods 

### 2.1. Prophage Identification

The roseobacter group was initially characterized as a clade; however, a recent phylogenomic analysis showed that the group is not monophyletic [59]. Results have shown six monophyletic clades containing members of the initial roseobacter group and a seventh clade that was not considered roseobacter [59]. Therefore, roseobacter (not capitalized or italicized) is used here not as a taxonomic group, but as an operational term referring to the *Rhodobacteraceae* that were historically classified as members of the *Roseobacter* clade. We selected 79 genomes of bacterial species belonging to 36 genera that are representative of the seven *Rhodobacteraceae* clades to investigate the genomics and distribution of prophages across the group. Complete genomes were retrieved from the NCBI RefSeq (RefSeq accession numbers, genome length, and number of contigs for each genome are reported in Table S1). We double-checked genome completion using CheckM [60]. All but one (*Rubellimicrobium thermophilum*, which is 87% complete) of the 79 genomes had more than 90% completion. Prophages encoded in these genomes were identified and annotated using VIBRANT (Virus Identification by IteRative ANnoTation) using default parameters (minimum scaffold length 1000 bp, minimum of four open reading frames) [61]. The bacterial genome files in FASTA format were supplied to VIBRANT, which performs Hidden Markov Model (HMM) searches against the Pfam, KEGG, and VOG databases [62,63,64]. VIBRANT identified 173 putative prophages based on the presence of viral hallmark genes: 5 as complete, 17 as high-quality drafts, 28 as medium-quality, and 123 as low-quality drafts (Table S2). The nucleotide sequences of the predicted prophages are available on FigShare (deposited on 10 April 2021). The contribution of prophages to the total bacterial genome (herein referred to as prophage density) was calculated by summing the genome length of all prophages in a given bacterial genome and dividing the result by the host genome length (Table S1). This analysis was performed separately for high- and medium-quality prophages and for all prophages identified.

### 2.2. Prophage Taxonomy and Phylogenomics

Putative prophages were dereplicated by clustering nucleotide sequences using CD-HIT at 95% identity [65]. Three approaches were utilized for prophage classification. First, gene annotations with high similarity to reference phages according to VIBRANT annotations were used to assign putative Lambda-like, Mu-like, other *Caudovirales*, and *Microviridae* prophages. Second, all predicted phage sequences were compared with the NCBI RefSeq viral database (10,003 genomes, accessed and downloaded 18 September 2020) using tBLASTx (E-value ≤ 10^−5^). The nucleotide sequences of high- and medium-quality phages were also compared to the JGI IMG Viral Database of cultivated and uncultivated viruses version 3.0 (>2,000,000 viral genome fragments, accessed on 4 January 2021) using the JGI web-based BLASTn tool (identity ≥ 30%, alignment length ≥ 90 bp, E-value ≤ 10^−5^) [66]. The IMG/VR 3 identified 31 viral contigs with similarities to the putative Roseobacter prophages. These sequences were combined with the 50 putative prophages with medium and high quality identified by VIBRANT for a phylogenomic analysis using the GL-UVAB workflow [67]. Briefly, the GL-UVAB database “Vir_DB_Nuc” (Accessed 16 November 2020) contained 195,698 reference (NCBI RefSeq) and metagenome-assembled viral sequences from 10 studies [67]. The database was filtered by excluding sequences shorter than 2 Kb in length and dereplicated at 98% identity, resulting in a database of 159,368 sequences [67,68]. Prodigal in metagenomic mode (-p meta) was used to predict protein encoding genes for both the GL-UVAB database and our predicted phages [69]. An all versus all amino acid comparison of the translated proteins was performed using DIAMOND (more sensitive mode, identity ≥ 30%, bitscore ≥ 30, alignment length ≥ 30 amino acids, and E-value ≤ 0.01) [70]. Contigs from the Vir_DB_Nuc sharing a minimum 3 proteins with putative prophages were extracted from the database and combined with the roseobacter prophage sequences for the phylogenomic analysis. Pairwise distances between genomes (putative roseobacter prophages and sequences from Vir_DB_Nuc and IMG/VR 3) were calculated using the Dice coefficient $D_{\mathrm{AB}}=1-\left(2\times \frac{\mathrm{AB}}{\mathrm{AA}+\mathrm{BB}}\right)$ where AB represents the sum of valid protein matches between sequences, and AA and BB are protein matches of sequences against themselves [67]. A tree was calculated from the pairwise Dice distances using the Phangorn package in R and visualized on the iTOL interactive web interface [71,72]. The original IDs of genomes and contigs included in the final tree, along with the information on the source database and isolation source, are described in Table S3.

### 2.3. Auxiliary Metabolic Genes and Gene Transfer Agents

Auxiliary metabolic genes identified by VIBRANT using the HMM searches against Pfam, KEGG, and VOG were binned into metabolic pathways by searching the Pfam accession of each gene in the KEGG database. The metabolic pathways of the genes were grouped by the prophage host genera (Table S4). The position of these genes in the phage genomes was visualized using EasyFig and Clinker [73,74]. Predicted prophages with the AMG *cysE*, the most abundant AMG identified in this dataset, were compared to each other and to the *Rhodobacter capsulatus* GTA sequence downloaded from the NCBI (GCA_000021865.1) using Clinker [74]. Clinker produces global alignments of amino acid sequences using the BLOSUM62 substitution matrix. The alignments were visualized with an identity threshold of 0.5.

### 2.4. Presence of Prophages in an Algae Bloom

Many roseobacters form symbiotic relationships with marine phytoplankton and macroalgae [13,75,76,77]. To test if the putative prophages identified were also observed in association with phytoplankton, we mapped metagenome reads from a dinoflagellate bloom against the 44 high- and medium-quality prophages identified here. The metagenomes were obtained from a previous study of a *Gymnodinium catenatum* dinoflagellate bloom in Shenzhen, China [78,79]. Metagenomes were generated by filtering water samples through a 10 μm filter followed by ${\mathrm{FeCl}}_{3}$ flocculation, 0.22 μm filtration, DNA extraction of the filters, and sequencing on an Illumina HiSeq 2000 (San Diego, CA, USA) [78]. Raw metagenome reads in FASTQ format were retrieved from the NCBI Sequence Read Archive (SRA) using the SRA toolkit (Accessed 16 December 2020). These viromes are YT1, YT3 (pre-bloom), YT4, YT5, YT6 (bloom), and YT8, YT19, and YT11 (post-bloom) [78]. The metagenomes were filtered in BBDuk, BBTools version 38.86 using quality trimming of both right and left sides (qtrim = rl), (trimq = 30), adapter trimming of both sides with a k-mer size of 23 (ktrim = l, ktrim = r, k = 23, mink = 11), a hamming distance of one (hdist = 1), and tpe and tpo parameters. Reads with an average quality below 30 (maq = 30), entropy below 0.90 (entropy = 0.90), and matches to PhiX were also removed [80]. Metagenome reads were mapped to the putative prophages using SMALT [81]. A SMALT index was created with a word length of 20 and a sampling step size of 10. The metagenomes were mapped to the index at 80% identity and only those prophage sequences recruiting at least 10 reads from at least one metagenome were kept in further analyses. The abundance of each prophage in the metagenome was visualized using the ANVI’O metagenomic workflow and using the “abundance” display mode in the interactive interface [82]. The abundances of algae and *Rhodobacterales* (as the closest approximation for the roseobacter group) in the seawater during the bloom were calculated in the original manuscript describing this algae bloom [79]. Briefly, dinoflagellate abundance was obtained by light microscopy counts. Bacterial cells stained with DAPI were quantified using an epifluorescence microscope. Abundance data were retried from the original manuscript using Webplot Digitizer version 4.4 (https://automeris.io/WebPlotDigitizer, accessed on 4 January 2021). To calculate *Rhodobacterales* abundance in cells/ml, the relative abundances from the metagenomes were multiplied by the total bacterial counts from epifluorescence microscopy.

### 2.5. Global Distribution of Roseobacter Prophages

We queried the TARA Oceans viromes for the presence of sequences closely related to the high- and medium-quality putative prophages identified here [83,84]. Raw reads from 200 TARA Oceans viral metagenomes (seawater filtered in 0.22 μm filters and concentrated using iron chloride flocculation) were mapped to our predicted prophages using Bowtie2 with the sensitive-local alignment mode [83,85,86]. The abundance of each prophage in the virome was expressed as reads per kilobase per million (RPKM) and visualized in R using the mapdata package [87]. The relative abundances of each prophage per virome were used as input for random forest analyses with 1000 permutations supervised by latitude, depth, temperature at 10 m depth, chlorophyll at 10 m depth, and chlorophyll at sample depth [88]. The mean decreasing error was used to rank important variables (prophages) with distributions significantly associated with latitude and temperature.

## 3. Results

### 3.1. Putative Prophages in Roseobacter Genomes

**Figure 1:**
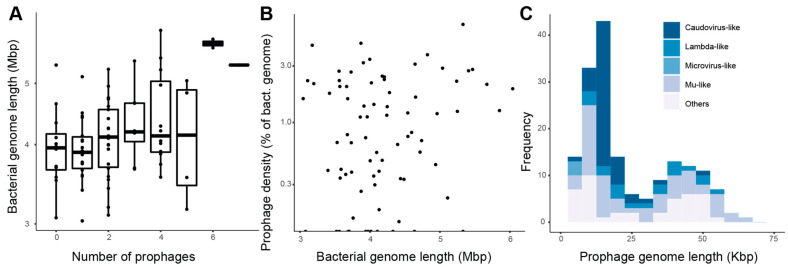
Prophages are widespread in roseobacter genomes. (**A**) Relationship between bacterial genome length and the number of putative prophages per genome. The horizontal line in the middle of each box represents the mean and the upper and lower bounds of the box represent the 75th and 25th percentiles. (**B**) Relationship between prophage density (sum of prophage lengths per each bacterial genome divided by bacterial genome length, plotted in log scale) and bacterial genome length (Linear regression *p* > 0.05). (**C**) Length frequency of all 173 putative prophages (Hartigans’ dip test for unimodality *p* > 0.05). Prophages are grouped by predicted taxonomy based on individual gene annotations.

The 79 roseobacter genomes analyzed here had an average length of 4.2 ± 0.076 Mbp (Mean ± SE). A total of 173 putative prophages were identified in these genomes by VIBRANT (5 complete, 17 high quality, 28 medium quality, and 123 low quality, Table S2), with at least one prophage identified in 66 roseobacter genomes. Five of the putative prophages had direct terminal repeats and formed complete circular genomes. Roseobacters had 2.19 ± 0.19 (Mean ± SE) prophages per genome (Figure 1A). If only high- and medium-quality prophages were taken into account, roseobacters had an average of 0.96 ± 0.14 (Mean ± SE) prophages per genome (Figure S1A). There was no relationship between the number of prophages and bacterial genome completeness (Linear regression *p* > 0.05). Roseobacter genome lengths were not significantly related to prophage length (Linear regression *p* > 0.05), and there was a weak relationship between the number of prophages and bacterial genome length (linear regression *p* = 0.00028, R^2^ = 0.158). Similar results were observed when analyzing the subset of high- and medium-quality predicted prophages (Figure 1A). The fraction of the total bacterial genome represented by putative prophages was defined as the prophage density. Prophage density was not significantly associated with the host genome length (Linear regression *p* > 0.05, Figure 1B), roseobacter clades as defined by Simon et al. 2017, isolation source (ANOVA *p* > 0.05 for both clades and isolation source), or host genome completeness (linear regression *p* > 0.05). Prophage length displayed a bimodal distribution (Hartigans’ dip test for unimodality *p* > 0.05, alternative hypothesis is at least bimodal) (Figure 1C). A taller peak was observed between 10 and 15 Kbp with a higher proportion of Caudovirus-like phages, and a smaller peak between 35 and 40 Kbp with a higher proportion of Mu-like and unidentified phages (Figure 1C). This pattern was different for the subset of high- and medium-quality prophages, which showed a unimodal distribution with a peak between 35 and 45 Kbp mostly comprised of Mu-like and unclassified phages (Hartigans’ dip test for unimodality *p* < 0.05, alternative hypothesis is at least bimodal) (Figure S1C).

**Figure 2:**
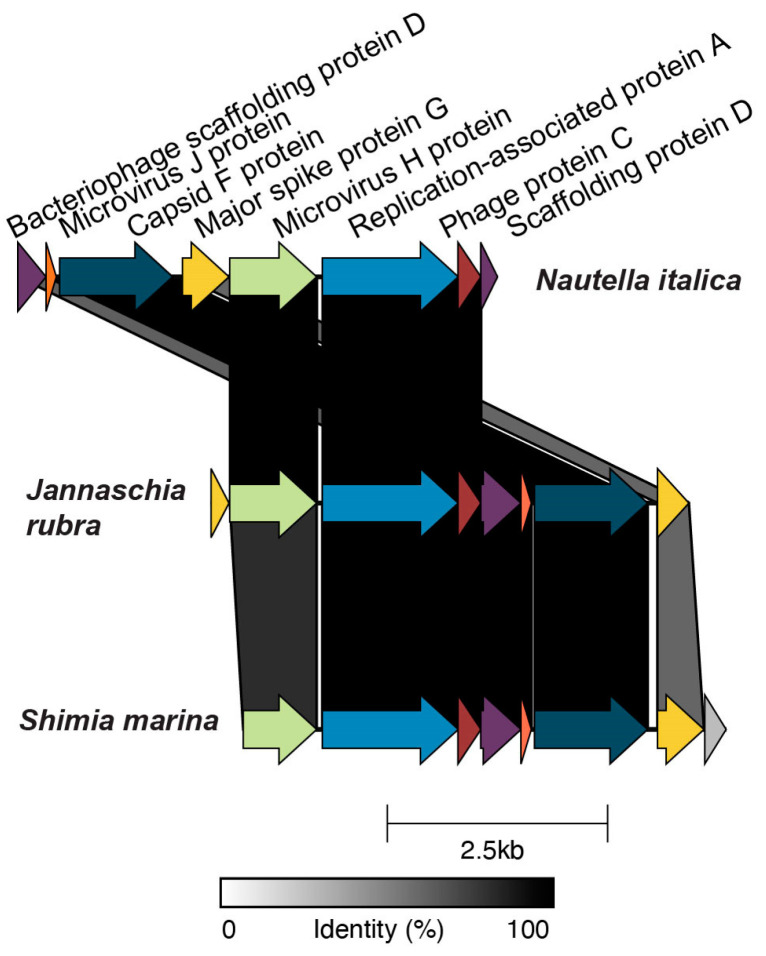
*Microviridae* prophages with a broad host range. The circular genomes are represented linearly, with the direction of the arrow representing the direction of transcription. Bacterial species names next to each prophage genome indicate the prophage hosts: *Nautella italica, Jannaschia rubra,* and *Shimia marina.* Alignment colors represent amino acid sequence identity (lower values for the microvirus H protein and the scaffolding protein D are due to differences in start and end positions in the original contigs identified through VIBRANT). Genome maps were plotted using Clinker [74].

Of the 50 high- or medium-quality prophages, 5 encoded an integrase, 18 a transposase, and 23 a recombinase. Eight prophages encoded no genes for integration and none of them encoded an excisionase. In total, 35% of the high- and medium-quality prophages were integrated into a tRNA gene, including tRNA-Gln, Leu, Ser, Gly, Thr, Val, Arg, Asn, and Met (Table S2). Three circular prophages were identified in five species, indicating complete genomes. The first, *Celeribacter halophilus* prophage 1, was 44,393 bp long, and the second, *Loktanella hongkongensis* prophage 1, was 17,661 bp long (Figure S2) and encoded a Caudovirus-like prohead serine protease (Pfam database, PF04586.17). No auxiliary metabolic genes were present in these two prophages. The third complete circular prophage was present in three roseobacter genomes with 100% sequence identity at the nucleotide level (BLASTn, E-value < 10^−5^) (Figure 2). The hosts for this prophage were *Jannaschia rubra*, *Shimia marina*, and *Nautella italica*, which belong to roseobacter clades 7, 1, and 1, respectively. In each host, the prophages had similar lengths, 5421 bp, 5478 bp, and 5441 bp. This prophage was classified as *Microviridae* and encoded three hallmark genes of *Microviridae*: the capsid protein (PF02305.17), the bacteriophage replication gene A protein (PF05840.13), and the microvirus J protein (PF04726.13) [89,90].

### 3.2. Auxiliary Metabolic Genes 

**Figure 3:**
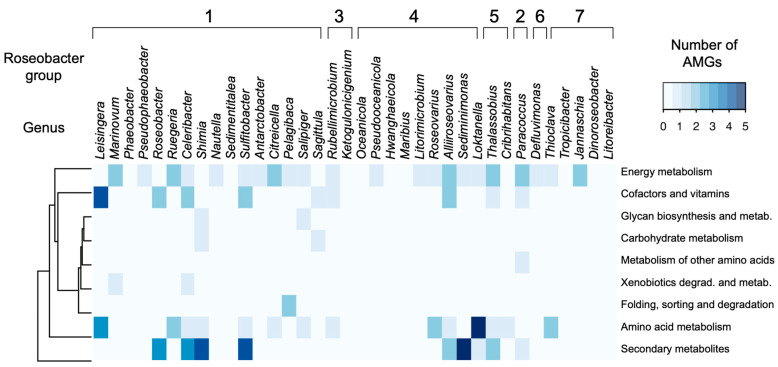
Roseobacter prophages encode auxiliary metabolic genes involved with secondary and amino acid metabolism. AMG distribution across the 36 roseobacter genera encoding prophages. Roseobacter genera are ordered and grouped according to their phylogenetic clade in Simon et al. 2017 [59].

Out of the 173 putative prophage regions identified in this study (including all low-, medium- and high-quality drafts), 61 encoded 98 auxiliary metabolic genes (Table S4). The AMGs were grouped into nine KEGG metabolic categories: amino acid metabolism, biosynthesis of secondary metabolites, carbohydrate metabolism, energy metabolism, folding, sorting and degradation, glycan biosynthesis, metabolism of cofactors and vitamins, metabolism of other amino acids, and xenobiotics biodegradation and metabolism (Figure 3). Energy metabolism, biosynthesis of secondary metabolites, and amino acid metabolism were the most abundant metabolic categories, with 28, 25, and 21 genes, respectively. The KEGG metabolic categories of the AMGs did not cluster by roseobacter clades (Figure 3). The gene *cysE* (serine O acetyltransferase) in the energy metabolism category was the most abundant AMG in the dataset, encoded by 22 phages. The gene *acpP* (acyl carrier protein) in the biosynthesis of secondary metabolites category was encoded by 8 phages, and *dcm* (DNMT1: DNA methyltransferase 1) in the amino acid metabolism category by 17 phages.

Three prophages—*Sulfitobacter delicatus* prophage 1, *Sulfitobacter dubious* prophage 2, and *Celeribacter indicus* prophage 2—encoded the three genes *acpP, fabF,* and *fabG* that are together involved in fatty acid synthesis. The gene *acpP* encodes an acyl carrier protein phosphopanthetine attachment site. The acyl carrier protein (ACP) carries the growing acyl chain for bacterial fatty acid synthesis, and is a target of the class of antimicrobial compounds pantothenate antimetabolites [91]. The gene *fabF* encodes 3-oxoacyl-[acyl carrier protein] synthase 2 and catalyzes the elongation of acyl-ACP [92]. The gene *fabG* encodes beta-ketoacyl-acyl carrier protein reductase and performs the first reductive step in the elongation of fatty acids [93]. The genomic organization for these three genes in all three phages from upstream to downstream is *fabG, acpP, fabF.* Other genes in the *fab* operon, *fabD* and *fabH,* were not present in any of the phages identified in our dataset.

### 3.3. Gene Transfer Agents

**Figure 4:**
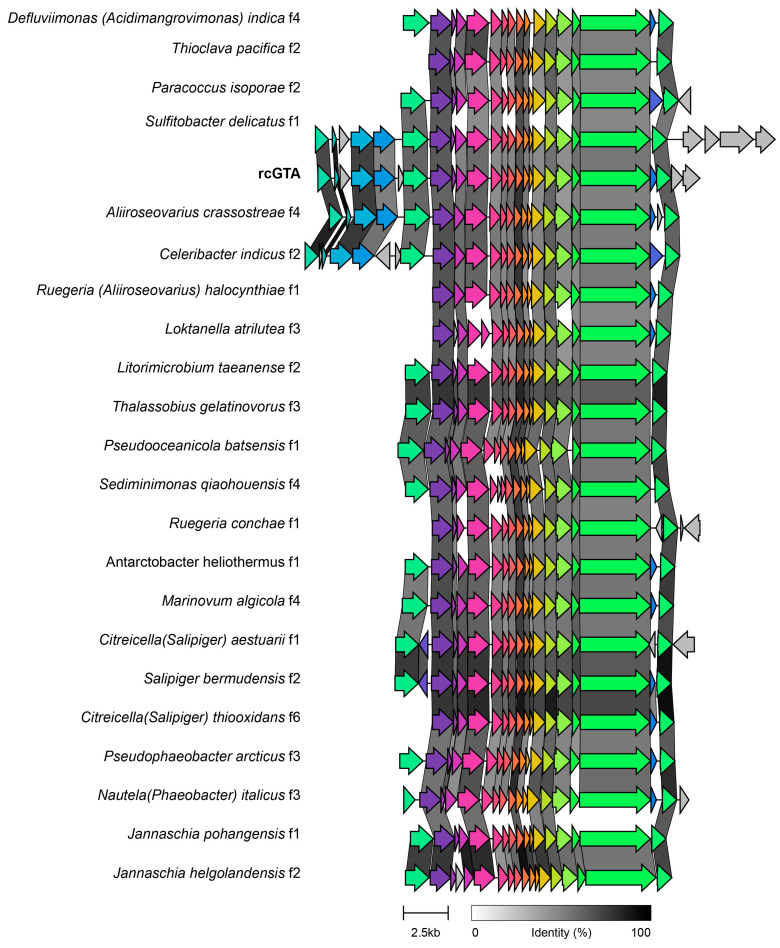
Genome alignments between the *Rhodobacter capsulatus* strain 1003 gene transfer agent (rcGTA, indicated in bold) and the 22 putative prophages encoding *cysE*. The grey bars indicate the percent identity of the amino acid alignments between shared genes. The letter *f* following the host name indicates genome fragment, which replaces “prophage” in this analysis.

The high prevalence of *cysE* genes in 22 putative prophages prompted further investigation. All the 22 putative prophages encoding this gene were classified as low-quality drafts by VIBRANT, and gene transfer agents (GTAs) in the neighborhood of *cysE* are highly conserved within *Rhodobacterales* [94]. To test if the 22 putative prophages were actually GTAs, we compared their genomes with that of a GTA of *Rhodobacter capsulatus* strain 1003, rcGTA. These genomic regions had high synteny and identity with the core gene cluster of rcGTA, from gene 1 to gene 15 (Figure 4). Therefore, these sequences were considered to be GTAs and were not included in subsequent analyses.

### 3.4. Prophage Phylogenomics

**Figure 5:**
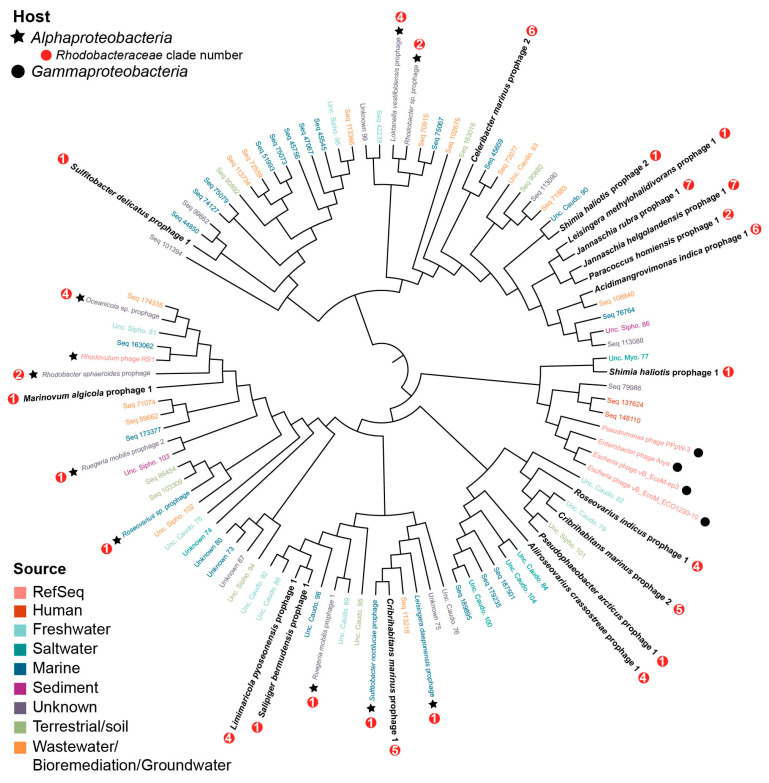
Roseobacter prophages represent novel viral groups. The tree shows in bold the 17 prophages identified in this study that had sequence similarities with the GL-UVAB and IMG/VR 3.0 viruses. The clade numbers of their roseobacter hosts (according to Simon et al., 2017) are labeled with a red circle. Among GL-UVAB and IMG/VR 3.0 viruses, the colors of sequence IDs indicate their environmental source. The star or circle indicates the predicted host. Leaf labels starting with Seq are from the GL-UVAB database, and leaves labeled Unknown represent IMG/VR database sequences (Table S3). For viruses with predicted taxonomy and/or predicted hosts, the lowest taxonomic level is shown.

A phylogenomics approach identified the closest relatives of the putative roseobacter prophages in the GL-UVAB [67] and IMG/VR databases. Only 17 of the putative roseobacter prophages shared at least three genes with viral contigs in both databases and were analyzed for phylogenomic relationships. An all-versus-all comparison of the genomes at the protein level generated a tree with two phylogenomic lineages. Both lineages contained viral genomes from marine ecosystems, freshwater, human samples, wastewater, groundwater, sediments, and terrestrial environments, with no clustering based on environmental source (Figure 5). The majority of hosts for the viral sequences obtained from the databases was unknown. Among the putative roseobacter prophages, sequences did not cluster based on roseobacter clades from Simon et al. 2017. *Shimia haliotis* prophage 1 was related to four *Myoviridae* phages infecting *Pseudomonas* and *Enterobacter* species (Enterobacter phage Arya, Escherichia phage vB_EcoM-ep3, Escherichia phage vB_EcoM_ECO1230-10, Pseudomonas phage PPpW-3) and uncultured myoviruses (Figure 5). The only other RefSeq genome related to the prophages identified here was *Rhodovulum* phage RS1. Despite being the closest known relatives of the roseobacter prophages, these RefSeq viruses were distant from other sequences in the tree (Figure 5). Other putative roseobacter prophages were related to five metagenome-assembled contigs, the putative hosts of which were also roseobacter: *Rhodobacter sphaeroides*, *Loktanella vestifoldensis*, *Rhodobacter* sp., *Oceanicola* sp., *Sulfitobacter noctilucae*, *Leisingera daeponensis*, *Roseovarius* sp., and *Rugeria mobilis* (Table S3).

### 3.5. Roseobacter Prophages in a Phytoplankton Bloom 

Roseobacters form mutualistic associations with marine phytoplankton and their abundances frequently follow that of dinoflagellate blooms [13,75,76,77,95]. To test if the putative roseobacter prophages identified here also associated with phytoplankton blooms, we searched for sequences with similarities to the roseobacter prophages in metagenomes from a bloom of the dinoflagellate *Gymnodinium catenatum* in the southern China Sea. Sequences with similarities to 17 of the high- and medium-quality putative roseobacter prophages were identified in the metagenomes obtained before, during, and after the bloom (Figure S3). The three most abundant phages in all eight metagenomes were encoded by *Shimia haliotis* (*Shimia haliotis* prophage 1 and prophage 2) and *Celeribacter marinus* (*Celeribacter marinus* prophage 2). Each of these prophages recruited a minimum of 1000 reads from each virome. However, prophage abundance in the metagenome did not have a relationship with roseobacter or dinoflagellate abundances through the progression of the bloom. *Shimia haliotis* prophage 1 contained a cluster of four auxiliary metabolic genes involved in the biosynthesis of secondary metabolites; *rfbA, rfbB, rfbC,* and *rfbD* (K01790, K10710, K00067, K00973). The *rfbABCD* cluster encodes a group of enzymes that synthesize the sugar dTDP-L-Rhamnose. Rhamnose is present in polysaccharides, glycoproteins, and O-antigen lipopolysaccharides. The fifth gene, identified as glycogen synthase, is involved in carbohydrate metabolism (K16150). *Celeribacter marinus* prophage 2 contained a single AMG, *acpP* (acyl carrier protein, phosphopanthetine attachment site). *Shimia haliotis* prophage 2 encoded no AMG.

### 3.6. Global Distribution of Roseobacter Prophages

**Figure 6:**
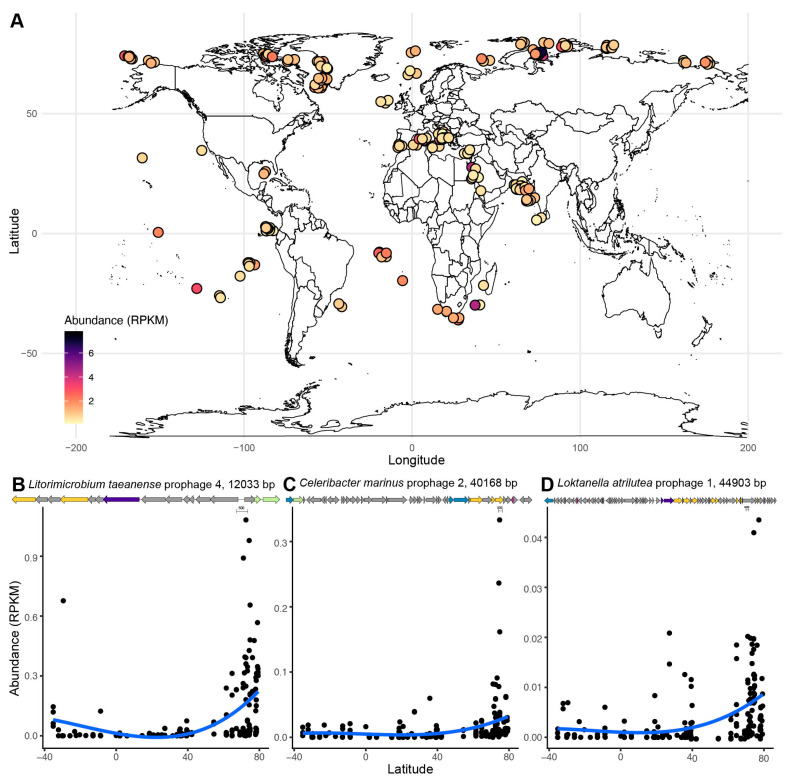
Roseobacter prophage distribution is significantly associated with latitude and temperature. Global distribution of high and medium quality predicted roseobacter prophages in 200 TARA Oceans viral metagenomes. (**A**) The abundance of each prophage in the virome was expressed as reads per kilobase per million (RPKM) and visualized in R using the mapdata package [87]. (**B**–**D**) Putative prophages significantly associated with both temperature and latitude according to a supervised random forest: *Litorimicrobium taeanense* prophage 4, *Celeribacter marinus* prophage 2, and *Loktanella atrilutea* prophage 1. The colors in the genome maps of these prophages indicate: structural genes in yellow, terminase in blue, integrase or recombinase in purple, AMG in pink, and unknown function in gray.

To investigate the global distribution of the putative roseobacter prophages identified here, reads with similarity to the 50 high- and medium-quality prophage sequences were searched in 200 viromes from the TARA Oceans Expeditions (Figure 6A). Sequences mapped to 41 of these prophages, and their relative abundances in the virome (in reads per kilobase per million), were significantly explained by latitude and temperature (Figure 6, supervised permutational random forest test, variability explained by temperature and latitude = 76.92 and 71.06%, respectively). The random forest models with depth, chlorophyll at 10 m depth, and chlorophyll at sample depth had low explanation power (26.21, 18.26, and 20.78%, respectively). The variable importance analyses from the supervised random forest identified the three putative prophages with the strongest relationship with both temperature and latitude (Figure 6B–D). The abundances of *Litorimicrobium taeanense* prophage 4, *Celeribacter marinus* prophage 2, and *Loktanella atrilutea* prophage 1 increased at higher latitudes. Both *Celeribacter marinus* prophage 2 and *Loktanella atrilutea* prophage 1 encode the AMG *acpP*, while *Litorimicrobium taeanense* prophage 4 does not encode any AMGs.

## 4. Discussion

### 4.1. Prophages Contribute to the Evolution of Roseobacter Genomes

Throughout its evolutionary history, the roseobacter group has undergone a net genome reduction with periods of genome expansion that included biased gene acquisition [96]. These gene acquisitions likely included prophage integration events followed by prophage domestication. The bimodal distribution of phage genome sizes observed here was consistent with previous results obtained from bacteria with similar genome length [1,97,98]. This bimodal distribution is likely a result of genetic degradation of the prophage sequences due to gene loss and purifying selection [2]. This process is known as prophage domestication by bacteria, and is a source of genetic novelty, contributing to a large fraction of the bacterial pangenomes [2]. The short putative prophage regions identified here are likely the result of these domestications. However, we found no evidence for a preferential retainment of auxiliary metabolic genes with ecologically or physiologically important functions for the host in the shorter putative prophages [99]. Likewise, no relationship between roseobacter genome length and prophage density (the fraction of the bacterial genome encoded in prophages) was observed (Figure 1B). These results contrast with a positive relationship between genome length and prophage density in bacteria with genomes up to 5.5 Mb in length [6].

### 4.2. Roseobacter Prophages Have Narrow Host Range

Most phages have narrow host ranges, with some exceptions, such as T4-like cyanophages that infect more than one genus [100]. Here, only one putative prophage was observed in different roseobacter species, indicating that most of these phages are specialists. This is consistent with the infection patterns of isolated roseophages, most of which only infect their original host strain [40]. The only exception to phage specialism identified in this study was the complete circular *Microviridae* prophage found in the genomes of three roseobacter species belonging to two clades, *Jannaschia rubra* (clade 7)*, Shimia marina* (clade 1)*,* and *Nautella italica* (clade 1) (Figure 2) [59]. Prophages belonging to *Microviridae* have been found in other bacteria, including a marine alphaproteobacterium [101,102]. The microvirus identified here does not belong to the genus *Gokushovirinae* within the *Microviridae,* previously described in roseobacters [89,90]. *Microviridae* contain three core genes, the major capsid protein, the minor spike protein (pilot protein), and the replication initiation protein, typified in the *Microviridae* ΦX174 [89,90,103]. The generalism in this broad host range prophage could have been selected due to intraspecific competition for bacterial hosts or conserved cell surface targets for phage infection [104,105].

### 4.3. Auxiliary Metabolic Genes and Gene Transfer Agents 

Among the 98 auxiliary metabolic genes identified here, none were consistently present across roseobacter genera or clades (Figure 3). Previously identified roseophage AMGs, such as thioredoxin genes (*trx*) and ribonucleoside triphosphate reductase (*rnr*), were not present in our dataset [23,44,46]. In a study categorizing the AMGs of isolated roseophages, seven genes were identified at high frequency: *trx, grx,* RNR, *thyX,* DCD, *phoH,* and *mazG* [106]. These AMGs are more frequent in lytic roseophages than those containing integrase, transposase, or repressor domains [106]. The putative prophages identified here did not encode these AMGs, suggesting that the previously described high-frequency genes are typical of lytic roseophages.

Three prophages in the genomes of *Sulfitobacter delicatus, Sulfitobacter dubius,* and *Celeribacter indicus* encode the genes *acpP, fabF,* and *fabG.* These three genes are together responsible for steps in the synthesis of fatty acids and phospholipids [107]. The gene *acpP* (acyl carrier protein) carries fatty acyl intermediates during fatty acid elongation, and *fabF* and *fabG* catalyze a condensation and reduction reaction in the elongation steps of synthesis, respectively [108]. In *E.* *coli* and *Vibrio harveyi,* the genes *fabG* and *fabF* are upstream and downstream of the gene *acpP,* respectively [107,109]. The genomic position in the three phages that encode all three genes *acpP, fabF,* and *fabG* is the same as that in *E. coli,* suggesting a conserved organization. The presence of this gene cluster in roseophages indicates that viruses are involved in fatty acid metabolism, analogously to the role of the fatty acid desaturases encoded by cyanophages [110].

The most common AMG in the predicted prophages, cysE, flanks a gene transfer agent (GTA) genomic region in Rhodobacter capsulatus, Rhodobacter sphaeroides, Roseobacter dentrificans, Oceanicola granulosus, *Roseobacter* sp. and Loktanella vestifoldensis [111]. The comparison between the Rhodobacter capsulatus GTA, rcGTA, with all putative prophage regions encoding cysE, revealed that these genomic regions have high synteny and homology to rcGTA (Figure 4) [94]. These results suggest that these 22 low-quality drafts initially identified as putative prophages are actually GTAs.

### 4.4. Roseobacter Prophages Represent Novel Viral Groups

*Shimia haliotis* prophage 1 was related to four *Myoviridae* phages infecting *Pseudomonas* and *Enterobacter* species and uncultured myoviruses (Figure 5). Two of these phages, PPpW-3 and Arya, encode integrases, but attempts to lysogenize the host in the lab were unsuccessful [112,113]. The other two phages, ECO1230-10 and EcoM-ep3, do not encode integrases. These results and the lack of repressor genes lead to the proposal that this myovirus group is strictly lytic and received the integrase gene through lateral gene transfer [113]. The presence of the *Shimia haliotis* prophage 1 integrated in the host genome indicated the ability for lysogeny within this group (Figure 5). Aside from the four *Myoviridae* phages related to *Shimia haliotis* prophage 1, the rest of the uncultivated contigs for which taxonomic identification was available belonged to the order *Caudovirales*.

The prophages identified in this study do not exhibit the genetic composition of the recently proposed Cobavirus group within *Podoviridae* [44]. This group is composed of two lytic phages infecting *Lentibacter* sp., Celeribacter marinus phage P12053L, *Roseobacter* sp. phage SIO1, and metagenome-assembled viral contigs [44]. Cobaviruses are characterized by a cobalamin-dependent ribonucleotide reductase (RNR) and a genomic organization into two arms, with replication genes to the left, and virion structure and morphogenesis on the right. The prophage sequences identified here do not display this organization or possess the cobalamin-dependent RNR.

### 4.5. Roseobacter Prophages Are Abundant in a Phytoplankton Bloom

Symbiotic interactions between roseobacter and microalgae are mediated by the exchange of metabolites, such as vitamin B_12_, nitrogen, DMSP, and carbon, and the ability of roseobacters to form biofilms on the algae surface [13,14,114,115]. By altering roseobacter metabolism, prophage-encoded AMGs may modulate chemical interactions that mediate these symbioses [114]. The presence of sequences with high similarity to the roseobacter prophages in metagenomes from a dinoflagellate bloom indicates that these prophages, or closely related temperate phages, are present in roseobacters that associate with phytoplankton (Figure S3). The most abundant of these prophages also encoded the gene *acpP* involved in type II fatty acid synthesis [116]. The second most abundant prophage in the bloom (host *Shimia haliotis*) encoded the gene cluster *rfbABCD* (Figure S3). These four highly conserved genes in Gram-positive and Gram-negative bacteria (also designated *rml* in Gram-positive bacteria) are involved in the synthesis of dTDP-rhamnose, a precursor to lipopolysaccharides, capsular polysaccharides, and exopolysaccharides [117,118]. The expression of *rfbABCD* complements O-antigen production mutation in *E. coli* [117]. Rhamnose is one of the sugar components of the O-repeating unit of the *E. coli* lipopolysaccharide, and a mutation in the *rfb* operon leads to loss of the O-antigen [119,120]. The O-antigen is used by some tailed bacteriophages as a receptor for attachment and infection [121]. In the roseobacter *Phaeobacter inhibens,* these four genes of the rhamnose operon are crucial for biofilm formation, a necessary step in the establishment of algal symbioses [75]. The genes forming the rhamnose operon in roseobacters are typically found in a RepA-I plasmid [22,75]. Loss of the extrachromosomal plasmid containing the rhamnose operon in *P. inhibens* leaves the bacteria unable to colonize the green algae *Ulva lactuca* [22]. The rhamnose operon identified here in a prophage in *Shimia haliotis* potentially implicates this prophage in the biofilm formation capabilities necessary for microalgae symbiosis.

### 4.6. The Global Distribution of Roseobacter Prophages Is Associated with Latitude

Roseophages are highly abundant in temperate and polar oceans, mirroring the distribution of their hosts [122,123,124,125,126]. Specifically, RCA, cobavirus and N4-like phage-infecting roseobacters increase in abundance in coastal environments [23,46,127]. Here, latitude and temperature were significantly associated with the distribution of roseobacter prophages in the TARA Oceans viromes (Figure 6). The high abundance of these prophages in the viromes from free viral particles suggests that they may be capable of inducing the lytic cycle in their hosts [128]. The three phages with the strongest relationship to latitude were *Litorimicrobium taeanense* prophage 4, *Celeribacter marinus* prophage 2, and *Loktanella atrilutea* prophage 1. Both *Loktanella atrilutea* prophage 1 and *Celeribacter marinus* prophage 2 contain the AMG *acpP,* which could be involved in regulating lipid membrane fluidity in polar temperatures [129].

## 5. Conclusions

The diversity of predicted prophages and gene transfer agents identified here in the genomes of roseobacters suggests that prophages and GTAs are a significant source of genomic diversity in this bacterial group. The auxiliary metabolic genes encoded by these prophages with functions in fatty acid metabolism and secondary metabolites are likely involved in the symbioses of their roseophage hosts with primary producers. Of particular importance may be the genes involved in fatty acid metabolism and carbohydrate modification, which may be involved in biofilm formation and nutritional exchange. The high abundance of some of these prophage sequences in the TARA Oceans viromes indicates that many of them may represent active prophages capable of entering the lytic cycle. The data presented here highlight the need for future studies on the metabolic changes incurred by prophage integration in roseobacter genomes.

## Data Availability

The nucleotide sequences of the predicted prophages are available on FigShare (https://doi.org/10.6084/m9.figshare.14397998.v1) (accessed on 10 April 2021). The NCBI accession numbers of the bacterial genomes analyzed are provided in Table S1.

## Supplementary Materials

The following are available online at https://www.mdpi.com/article/10.3390/microorganisms9061115/s1, Table S1: Bacterial genomes; Table S2: Prophages; Table S3: Phylogenomics; Table S4: AMGs; Figure S1: (**A**) Bacterial genome length plotted against the number of high and medium quality prophages identified in each of the 79 roseobacter genomes. The horizontal line in each box represents the mean, and the upper and lower bounds of the box represent the 75th and 25th percentiles. (**B**) Prophage density (total phage genome length divided by bacterial genome length) plotted against bacterial genome length. There is no correlation between the two variables (Pearson test *p* = 0.522). (**C**) Frequency of genome lengths for all high and medium quality prophages. The genome frequency is at least bimodal by Hartigans’ dip test for unimodality (*p* > 0.05). Each phage genome is grouped by color for predicted taxonomic assignments; Figure S2. (A) Genome of the predicted complete circular prophage from host *Celeribacter halophilus*. (B) Genome of predicted complete circular prophage from *Loktanella hongkongensis*. The circular genomes are represented linearly, with the direction of the arrow representing the direction of transcription. Figure S3: The abundance of high and medium quality prophages mapped at 80 % identity to eight G. catenatum algal bloom metagenomes from Du et al., and Huang et al [78,79]. Phages with greater than ten reads mapped in a metagenome are represented here. The color bar on the left-hand side denotes each phage. Black bars represent the phage abundance in each metagenome. The abundances of algae and Rhodobacterales at each time point were extracted from Huang et. al. 2018 [79]. The genomes of the three most abundant phages (recruiting more than 1,000 total reads) are shown on the right-hand side. These phages are labeled with their host and genome length, as well as the gene names for auxiliary meta-bolic genes. The mapped reads were visualized with ANVI’O version 6.2 using the metagenomic workflow using the abundance visualization, and the genome maps were created with EasyFig version 2.2.2.

## References

[1] Bobay L.-M., Touchon M., Rocha E.P.C. (2014). Pervasive domestication of defective prophages by bacteria. Proc. Natl. Acad. Sci. USA.

[2] Bobay L.-M., Rocha E.P.C., Touchon M. (2013). The adaptation of temperate bacteriophages to their host genomes. Mol. Biol. Evol..

[3] Casjens S. (2003). Prophages and bacterial genomics: What have we learned so far?. Mol. Microbiol..

[4] Zimmerman A.E., Howard-Varona C., Needham D.M., John S.G., Worden A.Z., Sullivan M.B., Waldbauer J.R., Coleman M.L. (2020). Metabolic and biogeochemical consequences of viral infection in aquatic ecosystems. Nat. Rev. Microbiol..

[5] Matos R.C., Lapaque N., Rigottier-Gois L., Debarbieux L., Meylheuc T., Gonzalez-Zorn B., Repoila F., de Fatima Lopes M., Serror P. (2013). Enterococcus faecalis Prophage Dynamics and Contributions to Pathogenic Traits. PLoS Genet..

[6] Touchon M., Bernheim A., Rocha E.P.C. (2016). Genetic and life-history traits associated with the distribution of prophages in bacteria. ISME J..

[7] Brüssow H., Canchaya C., Hardt W., Bru H. (2004). Phages and the evolution of bacterial pathogens: From genomic rearrangements to lysogenic conversion. Microbiol. Mol. Biol. Rev..

[8] Touchon M., De Sousa J.A.M., Rocha E.P.C. (2017). Embracing the enemy: The diversification of microbial gene repertoires by phage-mediated horizontal gene transfer. Curr. Opin. Microbiol..

[9] Luo H., Moran M.A. (2014). Evolutionary ecology of the marine Roseobacter clade. Microbiol. Mol. Biol. Rev..

[10] Buchan A., González J.M., Moran M.A. (2005). Overview of the Marine Roseobacter Lineage. Appl. Environ. Microbiol..

[11] Giebel H.-A., Brinkhoff T., Zwisler W., Selje N., Simon M. (2009). Distribution of Roseobacter RCA and SAR11 lineages and distinct bacterial communities from the subtropics to the Southern Ocean. Environ. Microbiol..

[12] Rappé M.S., Vergin K., Giovannoni S.J. (2000). Phylogenetic comparisons of a coastal bacterioplankton community with its counterparts in open ocean and freshwater systems. FEMS Microbiol. Ecol..

[13] Geng H., Belas R. (2010). Molecular mechanisms underlying roseobacter–phytoplankton symbioses. Curr. Opin. Biotechnol..

[14] Brinkhoff T., Bach G., Heidorn T., Liang L., Schlingloff A., Simon M. (2004). Antibiotic Production by a Roseobacter Clade-Affiliated Species from the German Wadden Sea and Its Antagonistic Effects on Indigenous Isolates. Appl. Environ. Microbiol..

[15] Lenk S., Moraru C., Hahnke S., Arnds J., Richter M., Kube M., Reinhardt R., Brinkhoff T., Harder J., Amann R. (2012). Roseobacter clade bacteria are abundant in coastal sediments and encode a novel combination of sulfur oxidation genes. ISME J..

[16] Sañudo-Wilhelmy S.A., Gómez-Consarnau L., Suffridge C., Webb E.A. (2014). The role of B vitamins in marine biogeochemistry. Ann. Rev. Mar. Sci..

[17] Helliwell K.E., Wheeler G.L., Leptos K.C., Goldstein R.E., Smith A.G. (2011). Insights into the evolution of vitamin B12 auxotrophy from sequenced algal genomes. Mol. Biol. Evol..

[18] Bertrand E.M., Allen A.E. (2012). Influence of vitamin B auxotrophy on nitrogen metabolism in eukaryotic phytoplankton. Front. Microbiol..

[19] Pujalte M.J., Lucena T., Ruvira M.A., Arahal D.R., Macián M.C., Rosenberg E., DeLong E.F., Lory S., Stackebrandt E., Thompson F. (2014). The Family Rhodobacteraceae. The Prokaryotes: Alphaproteobacteria and Betaproteobacteria.

[20] Harrison P.W., Lower R.P.J., Kim N.K.D., Young J.P.W. (2010). Introducing the bacterial ‘chromid’: Not a chromosome, not a plasmid. Trends Microbiol..

[21] Petersen J., Frank O., Göker M., Pradella S. (2013). Extrachromosomal, extraordinary and essential—The plasmids of the Roseobacter clade. Appl. Microbiol. Biotechnol..

[22] Frank O., Michael V., Päuker O., Boedeker C., Jogler C., Rohde M., Petersen J. (2015). Plasmid curing and the loss of grip—The 65-kb replicon of *Phaeobacter inhibens* DSM 17395 is required for biofilm formation, motility and the colonization of marine algae. Syst. Appl. Microbiol..

[23] Zhang Z., Chen F., Chu X., Zhang H., Luo H., Qin F., Zhai Z., Yang M., Sun J., Zhao Y. (2019). Diverse, Abundant, and Novel Viruses Infecting the Marine Roseobacter RCA Lineage. mSystems.

[24] Breitbart M., Bonnain C., Malki K., Sawaya N.A. (2018). Phage puppet masters of the marine microbial realm. Nat. Microbiol..

[25] Roux S., Brum J.R., Dutilh B.E., Sunagawa S., Duhaime M.B., Loy A., Poulos B.T., Solonenko N., Lara E., Poulain J. (2016). Ecogenomics and potential biogeochemical impacts of globally abundant ocean viruses. Nature.

[26] Kaneko H., Blanc-Mathieu R., Endo H., Chaffron S., Delmont T.O., Gaia M., Henry N., Hernández-Velázquez R., Nguyen C.H., Mamitsuka H. (2021). Eukaryotic virus composition can predict the efficiency of carbon export in the global ocean. iScience.

[27] Silveira C., Cavalcanti G., Walter J., Silva-Lima A., Dinsdale E., Bourne D., Thompson C., Thompson F. (2017). Microbial processes driving coral reef organic carbon flow. FEMS Microbiol. Rev..

[28] Knowles B., Silveira C.B., Bailey B.A., Barott K., Cantu V.A., Cobian-Guëmes A.G., Coutinho F.H., Dinsdale E.A., Felts B., Furby K.A. (2016). Lytic to temperate switching of viral communities. Nature.

[29] Silveira C.B., Rohwer F.L. (2016). Piggyback-the-Winner in host-associated microbial communities. NPJ Biofilms Microbiomes.

[30] Faruque S.M., Rahman M.M., Asadulghani, Islam K.M.N., Mekalanos J.J. (1999). Lysogenic Conversion of Environmental *Vibrio mimicus* Strains by CTXΦ. Infect. Immun..

[31] Silveira C.B., Coutinho F.H., Cavalcanti G.S., Benler S., Doane M.P.M.P., Dinsdale E.A., Edwards R.A., Francini-Filho R.B., Thompson C.C., Luque A. (2020). Genomic and ecological attributes of marine bacteriophages encoding bacterial virulence genes. BMC Genom..

[32] Steinberg K.M., Levin B.R. (2007). Grazing protozoa and the evolution of the *Escherichia coli* O157:H7 Shiga toxin-encoding prophage. Proc. R. Soc. B Biol. Sci..

[33] Rabinovich L., Sigal N., Borovok I., Nir-Paz R., Herskovits A.A. (2012). Prophage excision activates *Listeria* competence genes that promote phagosomal escape and virulence. Cell.

[34] Brüssow H. (2007). Bacteria between protists and phages: From antipredation strategies to the evolution of pathogenicity. Mol. Microbiol..

[35] Sunagawa S., Coelho L.P., Chaffron S., Kultima J.R., Labadie K., Salazar G., Djahanschiri B., Zeller G., Mende D.R., Alberti A. (2015). Structure and function of the global ocean microbiome. Science.

[36] Thompson L.R., Zeng Q., Kelly L., Huang K.H., Singer A.U., Stubbe J., Chisholm S.W. (2011). Phage auxiliary metabolic genes and the redirection of cyanobacterial host carbon metabolism. Proc. Natl. Acad. Sci. USA.

[37] Kieft K., Zhou Z., Anderson R.E., Buchan A., Campbell B.J., Hallam S., Hess M., Sullivan M.B., Walsh D.A., Roux S. (2020). Ecology of inorganic sulfur auxiliary metabolism in widespread bacteriophages. bioRxiv.

[38] Rohwer F., Segall A., Steward G., Seguritan V., Breitbart M., Wolven F., Azam F.F. (2000). The complete genomic sequence of the marine phage Roseophage SIO1 shares homology with nonmarine phages. Limnol. Oceanogr..

[39] Zhang Y., Zhang F., Yang J., Jiao N. (2012). Host responses of a marine bacterium, *Roseobacter denitrificans* OCh114, to phage infection. Arch. Microbiol..

[40] Zhan Y., Chen F. (2019). Bacteriophages that infect marine roseobacters: Genomics and ecology. Environ. Microbiol..

[41] Zhan Y., Huang S., Voget S., Simon M., Chen F. (2016). A novel roseobacter phage possesses features of podoviruses, siphoviruses, prophages and gene transfer agents. Sci. Rep..

[42] Huang S., Zhang Y., Chen F., Jiao N. (2011). Complete genome sequence of a marine roseophage provides evidence into the evolution of gene transfer agents in alphaproteobacteria. Virol. J..

[43] Ankrah N.Y.D., Budinoff C.R., Wilson W.H., Wilhelm S.W., Buchan A. (2014). Genome Sequence of the *Sulfitobacter* sp. Strain 2047-Infecting Lytic Phage ΦCB2047-B. Genome Announc..

[44] Bischoff V., Bunk B., Meier-Kolthoff J.P., Spröer C., Poehlein A., Dogs M., Nguyen M., Petersen J., Daniel R., Overmann J. (2019). Cobaviruses—A new globally distributed phage group infecting *Rhodobacteraceae* in marine ecosystems. ISME J..

[45] Li B., Zhang S., Long L., Huang S. (2016). Characterization and Complete Genome Sequences of Three N4-Like Roseobacter Phages Isolated from the South China Sea. Curr. Microbiol..

[46] Chan J.Z.-M., Millard A.D., Mann N.H., Schäfer H. (2014). Comparative genomics defines the core genome of the growing N4-like phage genus and identifies N4-like Roseophage specific genes. Front. Microbiol..

[47] Zhao Y., Wang K., Jiao N., Chen F. (2009). Genome sequences of two novel phages infecting marine roseobacters. Environ. Microbiol..

[48] Wittmann J., Turner D., Millard A.D., Mahadevan P., Kropinski A.M., Adriaenssens E.M. (2020). From Orphan Phage to a Proposed New Family–the Diversity of N4-Like Viruses. Antibiotics.

[49] Ceyssens P.-J., Brabban A., Rogge L., Lewis M.S., Pickard D., Goulding D., Dougan G., Noben J.-P., Kropinski A., Kutter E. (2010). Molecular and physiological analysis of three *Pseudomonas aeruginosa* phages belonging to the “N4-like viruses”. Virology.

[50] Angly F.E., Felts B., Breitbart M., Salamon P., Edwards R.A., Carlson C., Chan A.M., Haynes M., Kelley S., Liu H. (2006). The marine viromes of four oceanic regions. PLoS Biol..

[51] Sullivan M.B., Coleman M.L., Weigele P., Rohwer F., Chisholm S.W. (2005). Three *Prochlorococcus* Cyanophage Genomes: Signature Features and Ecological Interpretations. PLoS Biol..

[52] Yang Y., Cai L., Wang Y., Jiao N., Zhang R. (2018). Microarray analysis of gene expression of Dinoroseobacter shibae DFL12T in response to phage R2C infection. Mar. Genom..

[53] Ankrah N.Y.D., May A.L., Middleton J.L., Jones D.R., Hadden M.K., Gooding J.R., LeCleir G.R., Wilhelm S.W., Campagna S.R., Buchan A. (2014). Phage infection of an environmentally relevant marine bacterium alters host metabolism and lysate composition. ISME J..

[54] Ankrah N.Y.D., Budinoff C.R., Wilson W.H., Wilhelm S.W., Buchan A. (2014). Genome Sequences of Two Temperate Phages, CB2047-A and CB2047-C, Infecting *Sulfitobacter* sp. Strain 2047. Genome Announc..

[55] Zhao Y., Wang K., Ackermann H.-W., Halden R.U., Jiao N., Chen F. (2010). Searching for a “Hidden” Prophage in a Marine Bacterium. Appl. Environ. Microbiol..

[56] Tang K., Lin D., Zheng Q., Liu K., Yang Y., Han Y., Jiao N. (2017). Genomic, proteomic and bioinformatic analysis of two temperate phages in Roseobacter clade bacteria isolated from the deep-sea water. BMC Genom..

[57] Basso J.T.R., Ankrah N.Y.D., Tuttle M.J., Grossman A.S., Sandaa R.-A., Buchan A. (2020). Genetically similar temperate phages form coalitions with their shared host that lead to niche-specific fitness effects. ISME J..

[58] Wemheuer B., Wemheuer F., Hollensteiner J., Meyer F.-D., Voget S., Daniel R. (2015). The green impact: Bacterioplankton response toward a phytoplankton spring bloom in the southern North Sea assessed by comparative metagenomic and metatranscriptomic approaches. Front. Microbiol..

[59] Simon M., Scheuner C., Meier-Kolthoff J.P., Brinkhoff T., Wagner-Döbler I., Ulbrich M., Klenk H.-P., Schomburg D., Petersen J., Göker M. (2017). Phylogenomics of *Rhodobacteraceae* reveals evolutionary adaptation to marine and non-marine habitats. ISME J..

[60] Parks D.H., Imelfort M., Skennerton C.T., Hugenholtz P., Tyson G.W. (2015). CheckM: Assessing the quality of microbial genomes recovered from isolates, single cells, and metagenomes. Genome Res..

[61] Kieft K., Zhou Z., Anantharaman K. (2020). VIBRANT: Automated recovery, annotation and curation of microbial viruses, and evaluation of viral community function from genomic sequences. Microbiome.

[62] Kanehisa M., Goto S. (2000). KEGG: Kyoto encyclopedia of genes and genomes. Nucleic Acids Res..

[63] Sonnhammer E.L.L., Eddy S.R., Durbin R. (1997). Pfam: A comprehensive database of protein domain families based on seed alignments. Proteins Struct. Funct. Bioinform..

[64] Grazziotin A.L., Koonin E.V., Kristensen D.M. (2017). Prokaryotic Virus Orthologous Groups (pVOGs): A resource for comparative genomics and protein family annotation. Nucleic Acids Res..

[65] Fu L., Niu B., Zhu Z., Wu S., Li W. (2012). CD-HIT: Accelerated for clustering the next-generation sequencing data. Bioinformatics.

[66] Roux S., Páez-Espino D., Chen I.-M.A., Palaniappan K., Ratner A., Chu K., Reddy T.B.K., Nayfach S., Schulz F., Call L. (2021). IMG/VR v3: An integrated ecological and evolutionary framework for interrogating genomes of uncultivated viruses. Nucleic Acids Res..

[67] Coutinho F.H., Edwards R.A., Rodríguez-Valera F. (2019). Charting the diversity of uncultured viruses of Archaea and Bacteria. BMC Biol..

[68] Shen W., Le S., Li Y., Hu F. (2016). SeqKit: A Cross-Platform and Ultrafast Toolkit for FASTA/Q File Manipulation. PLoS ONE.

[69] Hyatt D., Chen G.-L., LoCascio P.F., Land M.L., Larimer F.W., Hauser L.J. (2010). Prodigal: Prokaryotic gene recognition and translation initiation site identification. BMC Bioinform..

[70] Buchfink B., Xie C., Huson D.H. (2015). Fast and sensitive protein alignment using DIAMOND. Nat. Methods.

[71] Letunic I., Bork P. (2007). Interactive Tree Of Life (iTOL): An online tool for phylogenetic tree display and annotation. Bioinformatics.

[72] Schliep K., Potts A.J., Morrison D.A., Grimm G.W. (2017). Intertwining phylogenetic trees and networks. Methods Ecol. Evol..

[73] Sullivan M.J., Petty N.K., Beatson S.A. (2011). Easyfig: A genome comparison visualizer. Bioinformatics.

[74] Gilchrist C.L.M., Chooi Y.-H. (2021). clinker & clustermap.js: Automatic generation of gene cluster comparison figures. Bioinformatics.

[75] Michael V., Frank O., Bartling P., Scheuner C., Göker M., Brinkmann H., Petersen J. (2016). Biofilm plasmids with a rhamnose operon are widely distributed determinants of the ‘swim-or-stick’ lifestyle in roseobacters. ISME J..

[76] Miller T.R., Belas R. (2004). Dimethylsulfoniopropionate Metabolism by *Pfiesteria*-Associated *Roseobacter* spp.. Appl. Environ. Microbiol..

[77] Howard E.C., Henriksen J.R., Buchan A., Reisch C.R., Bürgmann H., Welsh R., Ye W., González J.M., Mace K., Joye S.B. (2006). Bacterial Taxa That Limit Sulfur Flux from the Ocean. Science.

[78] Du X.-P., Cai Z.-H., Zuo P., Meng F.-X., Zhu J.-M., Zhou J. (2020). Temporal Variability of Virioplankton during a *Gymnodinium catenatum* Algal Bloom. Microorganisms.

[79] Huang X., Zhu J., Cai Z., Lao Y., Jin H., Yu K., Zhang B., Zhou J. (2018). Profiles of quorum sensing (QS)-related sequences in phycospheric microorganisms during a marine dinoflagellate bloom, as determined by a metagenomic approach. Microbiol. Res..

[80] Bushnell B., Rood J., Singer E. (2017). BBMerge—Accurate paired shotgun read merging via overlap. PLoS ONE.

[81] Ponstingl H., Ning Z. (2010). SMALT—A new mapper for DNA sequencing reads. F1000 Posters.

[82] Eren A.M., Esen Ö.C., Quince C., Vineis J.H., Morrison H.G., Sogin M.L., Delmont T.O. (2015). Anvi’o: An advanced analysis and visualization platform for ‘omics data. PeerJ.

[83] Brum J.R., Ignacio-Espinoza J.C., Roux S., Doulcier G., Acinas S.G., Alberti A., Chaffron S., Cruaud C., de Vargas C., Gasol J.M. (2015). Patterns and ecological drivers of ocean viral communities. Science.

[84] Gregory A.C., Zayed A.A., Conceição-Neto N., Temperton B., Bolduc B., Alberti A., Ardyna M., Arkhipova K., Carmichael M., Cruaud C. (2019). Marine DNA Viral Macro- and Microdiversity from Pole to Pole. Cell.

[85] Langmead B., Wilks C., Antonescu V., Charles R. (2019). Scaling read aligners to hundreds of threads on general-purpose processors. Bioinformatics.

[86] Langmead B., Salzberg S.L. (2012). Fast gapped-read alignment with Bowtie 2. Nat. Methods.

[87] Brownrigg R. Mapdata: Extra Map Databases 2018. R Package Version 2.3.0. https://CRAN.R-project.org/package=mapdata.

[88] Liaw A., Wiener M. (2002). Classification and Regression by RandomForest. R News.

[89] Cherwa J.E., Fane B.A. (2011). Microviridae: Microviruses and Gokushoviruses. eLS.

[90] Roux S., Krupovic M., Poulet A., Debroas D., Enault F. (2012). Evolution and Diversity of the *Microviridae* Viral Family through a Collection of 81 New Complete Genomes Assembled from Virome Reads. PLoS ONE.

[91] Zhang Y.-M., Frank M.W., Virga K.G., Lee R.E., Rock C.O., Jackowski S. (2004). Acyl Carrier Protein Is a Cellular Target for the Antibacterial Action of the Pantothenamide Class of Pantothenate Antimetabolites. J. Biol. Chem..

[92] Edwards P., Nelsen J.S., Metz J.G., Dehesh K. (1997). Cloning of the *fabF* gene in an expression vector and in vitro characterization of recombinant *fabF* and *fabB* encoded enzymes from Escherichia coli. FEBS Lett..

[93] Lai C.-Y., Cronan J.E. (2004). Isolation and Characterization of β-Ketoacyl-Acyl Carrier Protein Reductase (fabG) Mutants of *Escherichia coli* and *Salmonella enterica* Serovar Typhimurium. J. Bacteriol..

[94] Shakya M., Soucy S.M., Zhaxybayeva O. (2017). Insights into origin and evolution of α-proteobacterial gene transfer agents. Virus Evol..

[95] González J.M., Simó R., Massana R., Covert J.S., Casamayor E.O., Pedrós-Alió C., Moran M.A. (2000). Bacterial Community Structure Associated with a Dimethylsulfoniopropionate-Producing North Atlantic Algal Bloom. Appl. Environ. Microbiol..

[96] Luo H., Csűros M., Hughes A.L., Moran M.A. (2013). Evolution of Divergent Life History Strategies in Marine Alphaproteobacteria. mBio.

[97] Brueggemann A.B., Harrold C.L., Rezaei Javan R., van Tonder A.J., McDonnell A.J., Edwards B.A. (2017). Pneumococcal prophages are diverse, but not without structure or history. Sci. Rep..

[98] Crispim J.S., Dias R.S., Vidigal P.M.P., de Sousa M.P., da Silva C.C., Santana M.F., de Paula S.O. (2018). Screening and characterization of prophages in *Desulfovibrio* genomes. Sci. Rep..

[99] Khan A., Burmeister A.R., Wahl L.M. (2020). Evolution along the parasitism-mutualism continuum determines the genetic repertoire of prophages. PLoS Comput. Biol..

[100] Dekel-Bird N.P., Sabehi G., Mosevitzky B., Lindell D. (2015). Host-dependent differences in abundance, composition and host range of cyanophages from the Red Sea. Environ. Microbiol..

[101] Zheng Q., Chen Q., Xu Y., Suttle C.A., Jiao N. (2018). A virus infecting marine photoheterotrophic Alphaproteobacteria (Citromicrobium spp.) Defines a new lineage of ssDNA viruses. Front. Microbiol..

[102] Krupovic M., Forterre P. (2011). Microviridae goes temperate: Microvirus-related proviruses reside in the genomes of Bacteroidetes. PLoS ONE.

[103] McKenna R., Ilag L.L., Rossmann M.G. (1994). Analysis of the single-stranded DNA bacteriophage phi X174, refined at a resolution of 3.0 A. J. Mol. Biol..

[104] Bono L.M., Gensel C.L., Pfennig D.W., Burch C.L. (2013). Competition and the origins of novelty: Experimental evolution of niche-width expansion in a virus. Biol. Lett..

[105] Bertozzi Silva J., Storms Z., Sauvageau D. (2016). Host receptors for bacteriophage adsorption. FEMS Microbiol. Lett..

[106] Huang X., Jiao N., Zhang R. (2021). The Genomic Content and Context of Auxiliary Metabolic Genes in Roseophages. Environ. Microbiol..

[107] Shen Z., Byers D.M. (1996). Isolation of *Vibrio harveyi* acyl carrier protein and the *fabG, acpP*, and *fabF* genes involved in fatty acid biosynthesis. J. Bacteriol..

[108] Kutchma A.J., Hoang T.T., Schweizer H.P. (1999). Characterization of a *Pseudomonas aeruginosa* Fatty Acid Biosynthetic Gene Cluster: Purification of Acyl Carrier Protein (ACP) and Malonyl-Coenzyme A:ACP Transacylase (*FabD*). J. Bacteriol..

[109] Rawlings M. (1991). The gene encoding Escherichia coli acyl carrier protein lies within a cluster of fatty acid biosynthetic genes. J. Biol. Chem..

[110] Roitman S., Hornung E., Flores-Uribe J., Sharon I., Feussner I., Béjà O. (2018). Cyanophage-encoded lipid desaturases: Oceanic distribution, diversity and function. ISME J..

[111] Lang A.S., Beatty J.T. (2007). Importance of widespread gene transfer agent genes in α-proteobacteria. Trends Microbiol..

[112] Kawato Y., Yasuike M., Nakamura Y., Shigenobu Y., Fujiwara A., Sano M., Nakai T. (2015). Complete Genome Sequence Analysis of Two Pseudomonas plecoglossicida Phages, Potential Therapeutic Agents. Appl. Environ. Microbiol..

[113] Tikhe C.V., Gissendanner C.R., Husseneder C. (2018). Whole-Genome Sequence of the Novel *Enterobacter* Bacteriophage Arya with an Integrase Pseudogene, Isolated from the Gut of the Formosan Subterranean Termite. Genome Announc..

[114] Fuentes J.L., Garbayo I., Cuaresma M., Montero Z., González-del-Valle M., Vílchez C. (2016). Impact of Microalgae-Bacteria Interactions on the Production of Algal Biomass and Associated Compounds. Mar. Drugs.

[115] Seyedsayamdost M.R., Carr G., Kolter R., Clardy J. (2011). Roseobacticides: Small Molecule Modulators of an Algal-Bacterial Symbiosis. J. Am. Chem. Soc..

[116] Wong H.C., Liu G., Zhang Y.-M., Rock C.O., Zheng J. (2002). The Solution Structure of Acyl Carrier Protein from *Mycobacterium tuberculosis*. J. Biol. Chem..

[117] Boels I.C., Beerthuyzen M.M., Kosters M.H.W., Van Kaauwen M.P.W., Kleerebezem M., de Vos W.M. (2004). Identification and Functional Characterization of the *Lactococcus lactis rfb* Operon, Required for dTDP-Rhamnose Biosynthesis. J. Bacteriol..

[118] Giraud M.-F., Naismith J.H. (2000). The rhamnose pathway. Curr. Opin. Struct. Biol..

[119] Marolda C.L., Valvano M.A. (1995). Genetic analysis of the dTDP-rhamnose biosynthesis region of the *Escherichia coli* VW187 (O7:K1) rfb gene cluster: Identification of functional homologs of *rfbB* and *rfbA* in the rff cluster and correct location of the *rffE* gene. J. Bacteriol..

[120] Liu D., Reeves P.R. (1994). Escherichia coli K12 regains its O antigen. Microbiology.

[121] Broeker N.K., Barbirz S. (2017). Not a barrier but a key: How bacteriophages exploit host’s O-antigen as an essential receptor to initiate infection. Mol. Microbiol..

[122] Hwang J., Park S.Y., Park M., Lee S., Lee T.-K. (2017). Seasonal Dynamics and Metagenomic Characterization of Marine Viruses in Goseong Bay, Korea. PLoS ONE.

[123] Cai L., Zhang R., He Y., Feng X., Jiao N. (2016). Metagenomic Analysis of Virioplankton of the Subtropical Jiulong River Estuary, China. Viruses.

[124] Liang Y., Wang L., Wang Z., Zhao J., Yang Q., Wang M., Yang K., Zhang L., Jiao N., Zhang Y. (2019). Metagenomic Analysis of the Diversity of DNA Viruses in the Surface and Deep Sea of the South China Sea. Front. Microbiol..

[125] Ghai R., Hernandez C.M., Picazo A., Mizuno C.M., Ininbergs K., Díez B., Valas R., DuPont C.L., McMahon K.D., Camacho A. (2012). Metagenomes of Mediterranean Coastal Lagoons. Sci. Rep..

[126] Selje N., Simon M., Brinkhoff T. (2004). A newly discovered Roseobacter cluster in temperate and polar oceans. Nature.

[127] Labonté J.M., Swan B.K., Poulos B., Luo H., Koren S., Hallam S.J., Sullivan M.B., Woyke T., Wommack K.E., Stepanauskas R. (2015). Single-cell genomics-based analysis of virus–host interactions in marine surface bacterioplankton. ISME J..

[128] Luo E., Eppley J.M., Romano A.E., Mende D.R., DeLong E.F. (2020). Double-stranded DNA virioplankton dynamics and reproductive strategies in the oligotrophic open ocean water column. ISME J..

[129] Srinivas T.N.R., Nageswara Rao S.S.S., Vishnu Vardhan Reddy P., Pratibha M.S., Sailaja B., Kavya B., Hara Kishore K., Begum Z., Singh S.M., Shivaji S. (2009). Bacterial Diversity and Bioprospecting for Cold-Active Lipases, Amylases and Proteases, from Culturable Bacteria of Kongsfjorden and Ny-Ålesund, Svalbard, Arctic. Curr. Microbiol..

