# Supplementary material for: Prophage Genomics and Ecology in the Family Rhodobacteraceae
Source: Microorganisms. 2021 May 21;9(6):1115. doi: 10.3390/microorganisms9061115 (PMC8224337; doi:10.3390/microorganisms9061115)
Supplement: Supplementary file 1 [file microorganisms-09-01115-s001.zip › microorganisms-1198625-supplementary/microorganisms-1198625 supp figures.pdf]

Supplementary Figures

Prophage genomics and ecology in the family *Rhodobacteraceae*

Kathryn Forcone, Felipe H. Coutinho, Giselle S. Cavalcanti, Cynthia Silveira

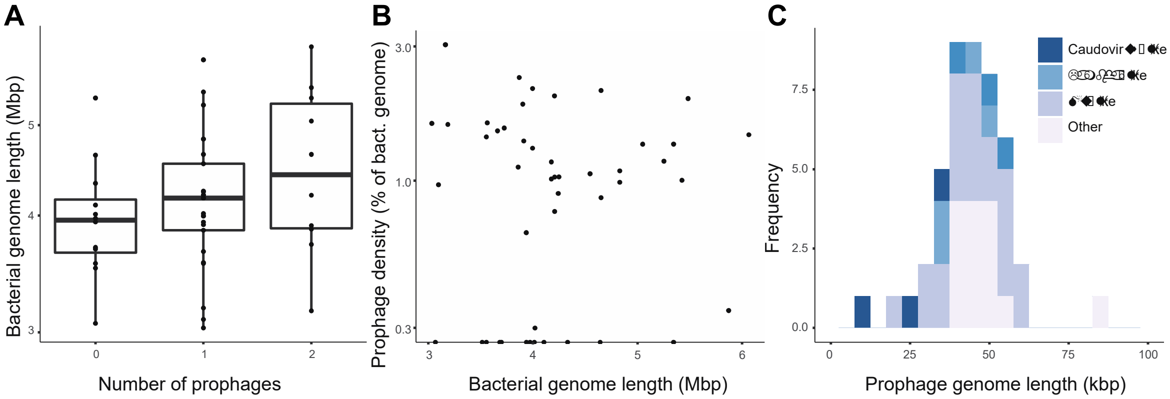

Figure S1. (**A**) Bacterial genome length plotted against the number of high and medium quality prophages identified in each of the 79 roseobacter genomes. The horizontal line in each box represents the mean, and the upper and lower bounds of the box represent the 75th and 25th percentiles. (**B**) Prophage density (total phage genome length divided by bacterial genome length) plotted against bacterial genome length. There is no correlation between the two variables (Pearson test $p = 0.522$). (**C**) Frequency of genome lengths for all high and medium quality prophages. The genome frequency is at least bimodal by Hartigans' dip test for unimodality ($p > 0.05$). Each phage genome is grouped by color for predicted taxonomic assignments.

.

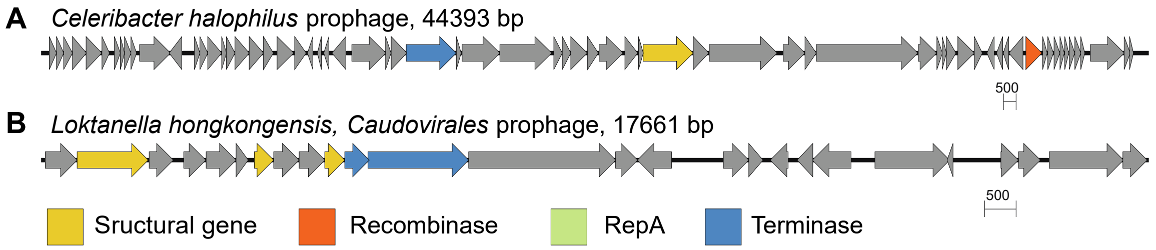

Figure S2. (**A**) Genome of the predicted complete circular prophage from host *Celeribacter halophilus.* (**B**) Genome of predicted complete circular prophage from *Loktanella hongkongensis.* The circular genomes are represented linearly, with the direction of the arrow representing the direction of transcription.

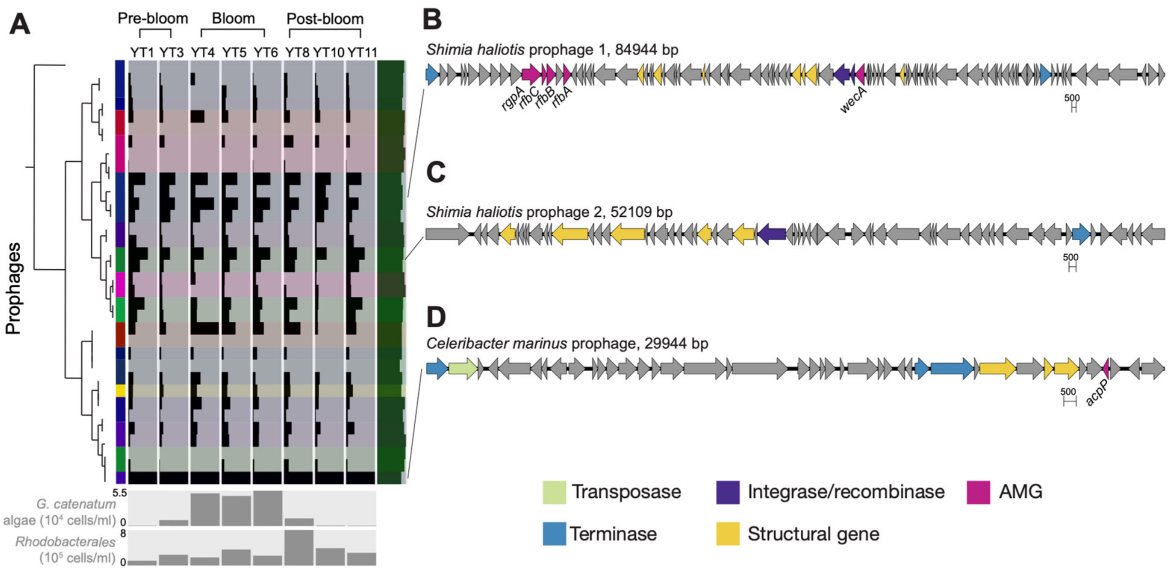

Figure S3. The abundance of high and medium quality prophages mapped at 80 % identity to eight *G. catenatum* algal bloom metagenomes from Du *et al.,* and Huang *et al.* [78,79]. Phages with greater than ten reads mapped in a metagenome are represented here. The color bar on the left-hand side denotes each phage. Black bars represent the phage abundance in each metagenome. The abundances of algae and *Rhodobacterales* at each time point were extracted from Huang et. al. 2018. [78,79]. The genomes of the three most abundant phages (recruiting more than 1,000 total reads) are shown on the right-hand side. These phages are labeled with their host and genome length, as well as the gene names for auxiliary metabolic genes. The mapped reads were visualized with ANVI'O version 6.2 using the metagenomic workflow using the abundance visualization, and the genome maps were created with EasyFig version 2.2.2.

78. Huang, X.; Zhu, J.; Cai, Z.; Lao, Y.; Jin, H.; Yu, K.; Zhang, B.; Zhou, J. Profiles of quorum sensing (QS)-related sequences in phycospheric microorganisms during a marine dinoflagellate bloom, as determined by a metagenomic approach. *Microbiol. Res.* **2018**, *217*, 1–13, doi:10.1016/j.micres.2018.08.015.
79. Du, X.-P.; Cai, Z.-H.; Zuo, P.; Meng, F.-X.; Zhu, J.-M.; Zhou, J. Temporal Variability of Virioplankton during a *Gymnodinium catenatum* Algal Bloom. *Microorganisms* **2020**, *8*, 107, doi:10.3390/microorganisms8010107.
